# Serum bilirubin levels and risk of type 2 diabetes: results from two independent cohorts in middle-aged and elderly Chinese

**DOI:** 10.1038/srep41338

**Published:** 2017-02-06

**Authors:** Jing Wang, Yaru Li, Xu Han, Hua Hu, Fei Wang, Xiulou Li, Kun Yang, Jing Yuan, Ping Yao, Xiaoping Miao, Sheng Wei, Youjie Wang, Weihong Cheng, Yuan Liang, Xiaomin Zhang, Huan Guo, Handong Yang, Jianmin Yuan, Woon-Puay Koh, Frank B. Hu, Tangchun Wu, An Pan, Meian He

**Affiliations:** 1 Department of Occupational and Environmental Health and State Key Laboratory of Environmental Health for Incubating, School of Public Health, Tongji Medical College, Huazhong University of Science and Technology, Wuhan, China; 2 Dongfeng Central Hospital, Dongfeng Motor Corporation and Hubei University of Medicine, Shiyan, Hubei, China; 3 Department of Epidemiology and Biostatistics, School of Public Health, Tongji Medical College, Huazhong University of Science and Technology, Wuhan, China; 4 Division of Cancer Control and Population Sciences, University of Pittsburgh Cancer Institute, Pittsburgh, PA, USA; 5 Department of Epidemiology, University of Pittsburgh Graduate School of Public Health, Pittsburgh, PA, USA; 6 Duke-NUS Medical School, Singapore, Singapore; 7 Department of Nutrition and Department of Epidemiology, Harvard T.H. Chan School of Public Health, Boston, MA, USA

## Abstract

Serum bilirubin is a potent endogenous antioxidant and has been identified as cardiovascular risk in cohort studies, while the relation to type 2 diabetes (T2D) in the elderly remains unclear. We investigated both cross-sectional and prospective associations between serum bilirubin levels and T2D risk in the Dongfeng-Tongji (DFTJ) cohort, and replicated the prospective findings in a nested case-control study (509 cases and 509 controls) within the Singapore Chinese Health Study (SCHS). In the cross-sectional analysis of DFTJ cohort (15,575 participants with 2,532 diabetes cases), serum bilirubin levels (total, direct and indirect) increased in new on-set diabetes and decreased with the diabetic duration. In the longitudinal analysis of DFTJ cohort (772 incident diabetes cases during 4.5 years of follow-up among 12,530 diabetes-free participants at baseline), positive association was found between direct bilirubin and T2D risk comparing extreme quartiles, similar results were observed in the nested case-control study within SCHS. Total and indirect bilirubin levels were not significantly associated with T2D in either cohort. In conclusion, our findings do not support the protective association between serum bilirubin levels and incident T2D in the middle-aged and elderly adults; instead, direct bilirubin levels were associated with increased risk of T2D.

Diabetes has become a serious public health concern worldwide, affecting more than 415 million people in 2015, and about one fourth of them were Chinese. More than 90% of diabetes cases are type 2 diabetes (T2D)^1^. Oxidative stress and inflammation have been implicated in the underlying pathogenesis^2^,^3^,^4^. Bilirubin, a potent antioxidant^5^,^6^, is one of the end products of heme catabolism in the system circulation, and shown to decrease the risk of cardiovascular disease in prospective studies^7^,^8^,^9^. Some cross-sectional studies have reported that bilirubin was negatively related to diabetic risk factors such as hypertension and metabolic syndrome^10^,^11^,^12^. Experimental studies in animal models suggested that bilirubin could protect beta cells from oxidative injury^13^,^14^ and enhance insulin sensitivity by decreasing oxidative stress and inflammation^15^,^16^,^17^.

However, in current cross-sectional studies^18^,^19^,^20^,^21^ and longitudinal studies^22^,^23^,^24^,^25^, the relation between bilirubin and dysglycemia remains controversial. Such discrepancies might be due to age differences. Serum bilirubin levels decreased with age in young adults^26^, and increased with age in middle-aged and elderly adults^18^,^27^. Moreover, previous studies mainly focused on serum total bilirubin (TBil)^18^,^19^,^20^,^22^,^23^,^24^,^25^, which is the sum of direct bilirubin (DBil) and indirect bilirubin (IBil). As traditional index of liver disease, TBil, DBil, and IBil have different clinical implications^28^. When TBil is in normal range, higher DBil may indicate hepatocellular injury^29^. Several studies had reported that DBil had more significant relationship to metabolic syndrome^21^,^30^ and stroke^31^ than TBil and IBil. Therefore, it is important to distinguish the temporal associations between different measures of bilirubin (TBil, DBil or IBil) and diabetic risk.

Therefore, we conducted the current analysis using data from the Dongfeng-Tongji (DFTJ) cohort, a prospective cohort study in a middle-aged and elderly Chinese population. We first examined the cross-sectional relation between serum bilirubin levels (TBil, DBil, and IBil) and prevalent diabetes, and then evaluated the prospective associations of bilirubin levels with incident diabetes during the 4.5 years of follow-up period. To validate our longitudinal results, we further replicated the analysis using data from a nested case-control study within the Singapore Chinese Health Study (SCHS).

## Results

The median (interquartile range) of serum TBil, DBil, and IBil levels was 13.3 (10.3–16.9), 3.7 (3.0–4.6), 9.5 (7.1–12.4) μmol/L, respectively. Baseline data according to the quartiles of TBil are presented in Table 1. Participants with higher serum TBil concentrations were more likely to be men, drinkers, never smokers, and with lower education levels. They also have elevated levels of AST, HDL, diastolic pressure, and lower levels of ALP (all *P*_trend_ < 0.05).

In the DFTJ cohort, a total of 772 T2D cases were identified during 4.5 year (56,723 person-years) of follow-up, corresponding to an incidence rate of 13.6 per 1000 person-years. As shown in the Table 2, compared with those in the lowest quartile of DBil levels, the HRs (95% CIs) were 1.22 (0.98–1.52), 1.39 (1.12–1.72), and 1.29 (1.03–1.61) for Q2–Q4 in the full adjusted model (*P*_trend_ = 0.03). No significant relationship was observed for TBil (*P*_trend_ = 0.27) or IBil (*P*_trend_ = 0.70). In sensitive analysis, the associations changed to null when we excluded the participants with impaired fasting glucose (IFG, those with relatively higher bilirubin levels and more likely to develop diabetes) at baseline (Supplementary Table S1).

We further validated the relation of bilirubin levels and risk of T2D in the SCHS (Table 3). In this cohort, the mean duration between blood donation and diagnosis of T2D was 4.0 (SD 1.7) years. DBil levels were positively associated with the risk of T2D, and the OR (95% CI) across tertiles was 1.00 (reference), 1.68 (1.14–2.47), and 1.63 (1.03–2.58), respectively (*P*_trend_ = 0.02) in the multivariate model. Similar to the DFTJ cohort, no significant relationship was observed for TBil (*P*_trend_ = 0.74) or IBil (*P*_trend_ = 0.86) with diabetes risk.

We further investigated the interaction between bilirubin levels and other covariates (sex, BMI, physical activity, drinking status, smoking status, and hypertension) on T2D risk in the DFTJ cohort. Although significant interactions were observed between smoking and bilirubin in the DFTJ cohort (Supplementary Figure S1), the interactions were not replicated in the SCHS (Supplementary Table S2).

**Figure 1:**
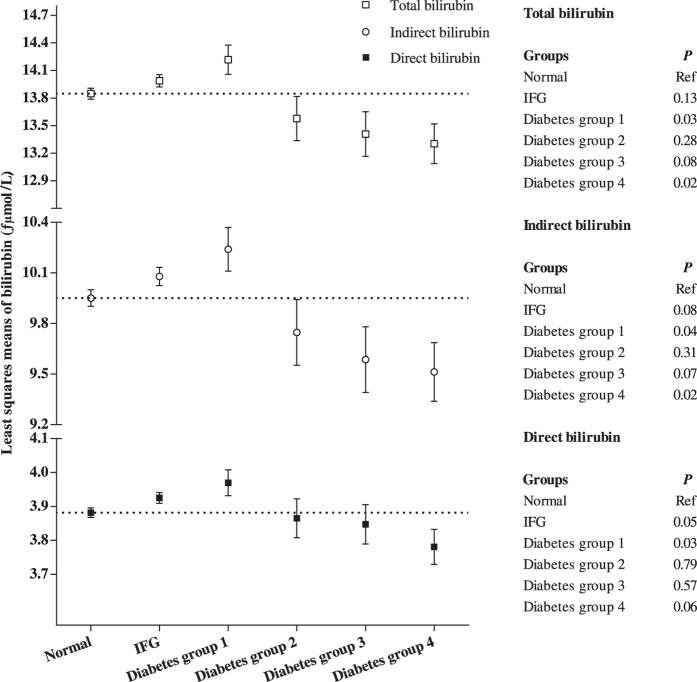
Least square means of three types of bilirubin levels in different groups. Least square means were adjusted for age, sex, BMI, waist circumference, education level, smoking status, drinking status, physical activity, family history of diabetes, history of hypertension, alkaline phosphatase, alanine aminotransferase, aspartate aminotransferase, triglyceride, high density lipoprotein and fasting plasma glucose. Black square = total bilirubin; white square = direct bilirubin; white circles = indirect bilirubin. Diabetes group 1: duration ≤1 year; Diabetes group 2: duration >1 year while ≤5 years; Diabetes group 3: duration >5 year while ≤10 years; Diabetes group 4: duration >10 years.

We additionally examined the associations of bilirubin levels with T2D risk in the cross-sectional design based on the baseline data of Dongfeng-Tongji cohort at 2008 (n = 15,575). As Table 4 showed, the serum TBil, DBil, and IBil levels were inversely associated with the risk of T2D (*P*_trend_ < 0.05) after adjustment for the traditional risk factors (model 1). Further adjustment for liver function and serum lipids diminished the associations to null. In addition, compared with the individuals with normal fasting glucose (n = 7,207), the IFG individuals (n = 5,836) had higher levels of bilirubin, and the new-onset diabetics (duration ≤ 1 year, n = 1,056, 41.2% of the diabetics) had the highest bilirubin levels (Fig. 1). In the next three diabetic groups (n = 454, 444 and 578, respectively), the bilirubin levels decreased with longer diabetic duration. Similarly, we found that the bilirubin levels were positively related to risk of T2D with 1-year duration but negatively with risk of T2D with more than 1-year duration (Supplementary Table S3).

## Discussion

In the present study, we found that elevated serum DBil concentrations were associated with an increased risk of developing T2D independent of traditional diabetes risk factors in two independent cohort studies of middle-aged and elderly Chinese adults. In contrast, no significant associations were found with total and indirect bilirubin levels. In addition, the serum bilirubin (TBil, DBil and IBil) levels were related to glucose metabolic status, and they increased in those with impaired fasting glucose and new-onset T2D, but decreased with the prolonged duration of diabetes.

Several cross-sectional studies have reported an inverse association between serum TBil and T2D^18^,^19^,^20^, while the results were not entirely consistent with some reporting no significant association between TBil and hyperglycemia^21^,^30^,^32^. Meanwhile, two cohort studies also reported an inverse association between TBil and T2D risk^24^,^25^. For example, a recent Mendelian randomization study in Dutch adults (n = 3,381) proposed that elevated serum TBil was causally associated with a decreased risk of T2D^24^. The other 4-year retrospective cohort study in Korean men (n = 5,960) indicated that high TBil levels were protective for the development of T2D^25^. In contrast, our study and the other two East Asian prospective studies^22^,^23^ did not find significant associate between TBil and T2D. Age and race might two main factors contribute to the inconsistent findings. Serum bilirubin levels are increased in older people^18^,^27^. The average age in our study was relatively older. In addition, serum bilirubin concentrations in East Asia populations^22^,^23^ are higher than those in whites^24^. In the Dutch population, 95% of the individuals had TBil concentrations lower than 10 μmol/L^24^; while in the present DFTJ cohort study, 75% of the individuals had TBil concentrations above 10 μmol/L. Therefore, the results in this European cohort study could not be directly generalized to East Asian population. In the Korean study which reported a protective association^25^, the concentrations of TBil (mean 20.1 μmol/L) were much higher than some other Korean studies^21^,^30^.

Notably, in the current study, we found that the DBil levels were associated with an increased but not decreased risk of incident T2D in two independent cohort studies of Chinese adults. Bilirubin (including TBil, IBil, and DBil) is traditional liver function index^28^. Serum IBil is carried by albumin to liver where the hepatic enzyme UDP-glucuronyl transferase 1A1 converts IBil to DBil. When TBil is in normal range, higher DBil may indicate hepatocellular injury^29^. Increased liver enzymes were related to the hepatic insulin resistance in healthy individuals^33^. Thus, the positive association of DBil levels with T2D risk might reflect the relation between liver dysfunction and T2D risk.

Evidence from studies about heme oxygenase (HO) system^34^,^35^ might also support the increased risk of bilirubin with T2D. Increased activity of HO could elevate the heme catabolic products such as carbo monoxide, iron, and bilirubin^34^. HO-1 has been reported as a strong positive predictor of metabolic inflammation among obese insulin-resistance individuals and animals^35^,^36^. In population studies, plasma HO-1 concentrations were elevated in impaired glucose regulation individuals and new-onset type 2 diabetic patients^37^,^38^. The higher bilirubin levels might be a biomarker of oxidative stress and inflammation in pre- and new-onset diabetes. While in individuals with overt diabetes, HO system might adapt to the long-term oxidative stress induced by hyperglycemia and result in a decreased expression of HO-1^39^. Meanwhile, reactive oxygen species generated by hyperglycemia in micro and macro vascular might lower the serum bilirubin concentrations. Therefore, long-term chronic hyperglycemia of diabetes might result in lower levels of serum bilirubin. Although bilirubin had been proven to be potent antioxidant^5^,^6^ and had the property of anti-inflammation^15^,^16^,^17^, the endogenous increased bilirubin levels are caused by the induction of HO through oxidative and inflammation reaction. More critically, we found serum bilirubin levels were influenced by glucose metabolic status: pre-diabetes and new-onset diabetes had higher bilirubin levels than individuals with normal fasting glucose, while the bilirubin levels decreased with the prolonged duration of diabetes (Fig. 1). This is consistent with a recent publication showing an inverse association between serum TBil and T2D duration^40^. This could partially explain why serum bilirubin levels were negatively associated with T2D risk in most cross-sectional studies^18^,^19^,^20^.

### Strengths and limitations

The strengths of the present study include both cross-sectional and longitudinal analyses base on the prospective cohort design and validation in another independent cohort; adjustment for multiple covariates including liver function; and evaluation of the bilirubin-diabetes association using TBil, DBil and IBil at the same time. More importantly, we found serum DBil was positively associated with incident T2D in two independent cohorts, indicating the robustness of the present results.

There are also several limitations of the present study. Firstly, although we speculate that the HO system played an important role in the bilirubin effects on T2D development, but we did not measure the levels of HO, and markers of inflammation and oxidative stress in the present population. However, previous studies have suggested that plasma HO-1 levels are positively associated with T2D risk^37^,^38^, lending support to the present findings. Secondly, the follow-up periods in DFTJ and SCHS cohorts were relatively short, and statistical power might be limited because of the small number of incident cases. Nonetheless, the effects of three type of bilirubin were similar in the two independent populations suggesting low probability of chance findings. Thirdly, we only measured serum bilirubin once at baseline, and the concentrations may not represent long-term exposure status. Finally, the current study was conducted in middle-aged and elderly Chinese population, and further studies with different ethnic and age populations are required to confirm our findings.

## Conclusions

In summary, we found serum DBil concentrations were positively associated with the risk of incident T2D in middle-aged and elderly adults. Additional large and long-term prospective studies in different ethnic groups are warranted to establish the role of serum bilirubin in the T2D development.

## Methods

### Study population

The DFTJ cohort is an ongoing dynamic prospective cohort including 27,009 retirees who come from the Dongfeng Motor Corporation with an average age of 63.6 years at study inception in 2008^41^. Each participant completed a semi-structured questionnaire including the socio-demographic, lifestyle, health status, and medical history, and also physical examination including fasting blood sample collection. Participants with available data of serum bilirubin and normal urobilirubin and urobilinogen were eligible for the present analysis (n = 23,482). We further excluded those with self-reported history of liver diseases and cancer (n = 1,633), abnormal levels of aspartate aminotransferase (AST) or alanine aminotransferase (ALT) or alkaline phosphatase (ALP) (defined as values above the 95% reference levels; n = 2,466), and missing data for fasting glucose (n = 7). To rule out those who might potentially have Gilber syndrome, subjects with TBil above 34.2 μmol/L were also excluded (n = 117)^42^. We further excluded subjects who reported to have coronary heart disease (CHD) or stroke (n = 3,684). The remained participants were 15,575. In 2013, a total of 14,973 eligible participants who attended the baseline survey were successfully followed. In the longitudinal analysis, we further excluded 2,443 individuals with diabetes at baseline, and 12,530 participants remained.

We further validated the results in a nested case-control study from the SCHS cohort. Details of the SCHS have been reported elsewhere^43^,^44^. Briefly, participants were all middle-aged and elderly Chinese in Singapore and free of diabetes at the time of blood collection between 1999 and 2004. They were contacted by telephone during the follow-up interview occurred in 2006–2010. A total of 509 incident diabetes cases were selected for the present analysis, and 509 controls were randomly selected from the remaining diabetes-free participants and matched on age (±3 years), sex, dialect group (Cantonese, or Hokkien), and date (±6 months) of blood collection. The detailed characteristics of the subjects in this nested case-control study were present in the Supplementary Table S4. All participants were free of CHD, stroke or cancer at baseline.

### Ethics statement

The DFTJ cohort study was approved by the Medical Ethics Committee of the School of Public Health, Tongji Medical College, and the Dongfeng General Hospital in the Dongfeng Motor Corporation. The SCHS cohort was approved by the Institutional Review Board of the National University of Singapore. The methods were carried out in accordance with the relevant guidelines. All subjects enrolled gave written informed consent for participation.

### Laboratory measurements

In the DFTJ cohort, peripheral venous blood samples were collected after overnight fasting. Plasma glucose levels were measured with Aeroset automatic analyzer (by glucose oxidase method; Abbott Laboratories. Abbott Park, Illinois, USA). The serum bilirubin, lipids, hepatic function, and renal function were measured by the ARCHITECTCi8200 automatic analyzer (ABBOTT Laboratories. Abbott Park, Illinois, USA). In the SCHS, morning random blood samples were collected, and plasma lipids, hepatic enzymes, and bilirubin were measured via colorimetric method on a chemistry analyzer (AU5800 Analyzer, Beckman Coulter, Brea, CA).

### T2D definition

In the DFTJ cohort, T2D was defined as having fasting plasma glucose (FPG) ≥7.0 mmol/L, according to the WHO criteria^45^, or having self-reported doctor-diagnosed diabetes or taking antidiabetic medications. In the SCHS, history of physician-diagnosed diabetes was asked at each follow-up interview by the question: “Have you been told by a doctor that you have diabetes?” If the answer was “yes”, participants were further asked for the age at which they were first diagnosed. In a validation study of the SCHS cohort participants, 97% of the self-reported diabetes cases were confirmed to be valid, suggesting a very high positive predictive value^46^.

### Statistical analysis

In the DFTJ cohort, baseline characteristics data were compared across quartiles of TBil. Categorical variables were expressed in percentages and continuous variables in mean (SD) or median (interquartile range). Covariate distributions across baseline TBil quartiles were compared using logistic regression models for categorical variables and ANOVA for continuous variables.

Follow-up time was calculated from baseline to the date of diagnosis of T2D, death, or the follow-up interview, whichever came first. Cox proportional hazards models were used to examine hazard ratio (HR) and confidence intervals (CIs) of T2D for each bilirubin quartile compared with the lowest quartile, with adjustment for age (continuous), sex, BMI (<24 and ≥24 kg/m^2^), central obesity (binary variable defined as waist circumference ≥85 cm in men or ≥80 cm in women), education (below secondary school, secondary school or higher), smoking status (ever and never smoker), drinking status (ever and never drinker), physical activity (yes, no), family history of diabetes (yes, no), baseline history of hypertension (yes, no). Liver function measures (ALT, AST, and ALP), triglyceride, and high density lipoprotein (HDL) were treated as continuous variables and included in the final model. To test the linear trend across bilirubin quartiles, we assigned the median value to each quartile, and treated it as continuous variable in the model. Stratified analyses were performed according to sex, BMI category, smoking, drinking, and history of hypertension. We also calculated the HRs associated with per 1 SD increase in bilirubin. Likelihood ratio tests were conducted to examine interactions.

In the cross-sectional analysis of Dongfeng-Tongji cohort, logistical regression models were used to examine Odds ratios (OR) and 95% CIs of T2D for three types of bilirubin. To investigate the change of bilirubin levels among different glucose metabolic groups (individuals with normal fasting glucose, impaired fasting glucose, diabetes with different duration), we used generalized linear models to calculate least squares means of bilirubin levels among different groups. According to diabetes duration, T2D cases were classified into four groups: duration ≤1 year; 1 year <duration ≤5 years; 5 years <duration ≤10 years; and duration >10 years.

In the nested case-control study from SCHS, participants were classified into three categories according to the levels of bilirubin because of relative small sample size and accuracy of the data (values were rounded to integral numbers from the lab). Conditional Logistic regression models were used to estimate ORs and 95% CIs. Covariates in model included age, BMI (<24 and ≥24 kg/m^2^), education (below secondary school, secondary school or higher), smoking status (ever and never smokers), drinking status (ever and never drinkers), physical activity levels (<0.5, ≥0.5 hours/week), continuous values of ALP, ALT, AST, triglyceride, and high density lipoprotein, baseline history of hypertension, and fasting status. All statistical analyses were performed with SAS version 9.4 (SAS Institute, Cary, North Carolina, USA). Two-sided *P* < 0.05 was considered as statistically significant.

## Additional Information

**How to cite this article**: Wang, J. *et al*. Serum bilirubin levels and risk of type 2 diabetes: results from two independent cohorts in middle-aged and elderly Chinese. *Sci. Rep.* **7**, 41338; doi: 10.1038/srep41338 (2017).

**Publisher's note:** Springer Nature remains neutral with regard to jurisdictional claims in published maps and institutional affiliations.

## Supplementary Material

Supplementary Tables and Figure

## Figures and Tables

**Table 1:** Baseline characteristics of the Dongfeng-Tongji cohort according to serum total bilirubin quartiles.

Characteristics	Total bilirubin (μmol/L)				*P* for trend
	Q1	Q2	Q3	Q4	
	2.8~	10.3~	13.3~	16.9~34.2	
N	3102	3168	3087	3173	—
Total bilirubin (μmol/L)	8.6 (7,9.4)	11.6 (11.1,12.3)	14.8 (14.0,15.7)	19.2 (18.3,22.9)	—
Direct bilirubin (μmol/L)	2.8 (2.5,3.3)	3.4 (3.1,3.8)	4.1 (3.5,4.5)	5.1 (4.3,5.9)	—
Indirect bilirubin (μmol/L)	5.8 (4.7,6.5)	8.2 (7.7,9)	10.7 (9.9,11.5)	14.3 (13.2,17.1)	—
Age, years	60.9 (7.8)	61.9 (7.8)	62.6 (7.6)	63 (7.4)	0.019
Sex, men(%)	28.8	36.8	46.8	57.6	<0.001
High school or above, (%)	36.7	35.4	34.8	32.9	<0.001
Smoker, (%)	21.7	25.2	29.6	35.1	<0.001
Drinker, (%)	19.4	23.6	28.3	34.4	0.004
Physical activity, (%)	87.7	89.4	90.3	89.8	0.032
FPG (mmol/L)	5.5 (0.6)	5.5 (0.6)	5.5 (0.6)	5.5 (0.6)	0.085
IFG (%)	43.4	43.2	45	47.3	0.18
ALP (U/L)	85 (69,102)	86 (72,102)	85 (70,101)	83 (69,98)	0.001
ALT (U/L)	19 (15,25)	19 (15,25)	19 (15,25)	20 (15,26)	0.45
AST (U/L)	22 (19,26)	22 (19,26)	23 (19,26)	23 (20,27)	<0.001
Total cholesterol (mmol/L)	5.2 (0.9)	5.2 (0.9)	5.2 (0.9)	5.1 (0.9)	0.89
Triglyceride (mmol/L)	1.2 (0.8,1.7)	1.2 (0.8,1.6)	1.1 (0.8,1.6)	1.1 (0.8,1.6)	0.057
High density lipoprotein (mmol/L)	1.4 (0.4)	1.5 (0.4)	1.5 (0.4)	1.5 (0.4)	<0.001
Low density lipoprotein (mmol/L)	3 (0.8)	3 (0.8)	3 (0.9)	3 (0.8)	0.021
Systolic pressure (mmHg)	126.2 (18.3)	127.3 (18.4)	127.4 (17.9)	128.3 (18.5)	0.19
Diastolic pressure (mmHg)	76.8 (10.5)	77.3 (10.9)	77.4 (10.8)	77.8 (10.8)	0.019
Overweight/obesity, (%)	48.7	50.7	50.4	50.7	0.61
Central obesity, (%)	44.4	41.4	39.3	37.6	0.67
Family history of diabetes, (%)	5.4	3.5	3.5	3.4	0.26
Hypertension, (%)	43.0	45.2	46	48.3	0.057

Data are means (SD), percentage (%), or median (interquartile range); *P*-value was calculated after adjustment for age, sex except for itself. FPG, fasting plasma glucose; IFG, impared fasting glucose; ALP, alkaline phosphatase; ALT, alanine aminotransferase; AST, aspartate aminotransferase.

**Table 2:** Associations of serum bilirubin levels and risk of type 2 diabetes incidence in Dongfeng-Tongji cohort (hazard ratio and 95% confidence interval).

		Quartiles of serum bilirubin (μmol/L)				*P* for trend
				Q1	Q2		Q3	Q4
**Total bilirubin**	Range	2.8~	10.3~	13.3~	16.9~34.2	
	Median	8.6	11.6	14.8	19.2	
	Cases/Person-years	185/14062.82	170/14371.89	224/13962.32	193/14326.08	
	Age-, sex- adjusted	1.00 (Ref)	0.91 (0.74–1.13)	1.22 (1.00–1.49)	1.03 (0.84–1.27)	0.31
	Model 1	1.00 (Ref)	0.90 (0.73–1.11)	1.21 (1.00–1.48)	1.02 (0.83–1.26)	0.34
	Model 2	1.00 (Ref)	0.92 (0.74–1.15)	1.28 (1.05–1.57)	1.04 (0.84–1.29)	0.27
**Direct bilirubin**	Range	0.8~	3.0~	3.7~	4.6~19.4	
	Median	2.6	3.3	4.1	5.3	
	Cases/Person-years	160/13090.16	190/14075.84	218/14923.01	204/14634.1	
	Age-, sex- adjusted	1.00 (Ref)	1.15 (0.93–1.42)	1.28 (1.04–1.58)	1.21 (0.98–1.50)	0.08
	Model 1	1.00 (Ref)	1.15 (0.93–1.43)	1.31 (1.06–1.61)	1.23 (0.99–1.52)	0.06
	Model 2	1.00 (Ref)	1.22 (0.98–1.52)	1.39 (1.12–1.72)	1.29 (1.03–1.61)	0.03
**Indirect bilirubin**	Range	1.1~	7.1~	9.5~	12.4~27.7	
	Median	5.7	8.1	10.7	14.3	
	Cases/Person-years	189/13865.8	170/14009.85	219/14627.99	194/14219.47	
	Age-, sex- adjusted	1.00 (Ref)	0.89 (0.73–1.10)	1.10 (0.90–1.34)	0.98 (0.80–1.20)	0.70
	Model 1	1.00 (Ref)	0.88 (0.71–1.09)	1.11 (0.91–1.35)	0.96 (0.78–1.18)	0.83
	Model 2	1.00 (Ref)	0.90 (0.73–1.11)	1.17 (0.96–1.43)	0.97 (0.79–1.20)	0.70

Model 1: adjusted for age, sex, body mass index, waist circumference, education level, smoking status, drinking status, physical activity, and family history of diabetes; Model 2: additionally adjusted for serum levels of alkaline phosphatase, alanine aminotransferase, aspartate aminotransferase, triglyceride, high density lipoprotein and history of hypertension.

**Table 3:** Associations of serum bilirubin levels and risk of type 2 diabetes in Singapore Chinese Health Study (Odds ratio and 95% confidence interval).

Bilirubin type		Tertiles of serum bilirubin levels (μmol/L)			*P* for trend
		T1	T2	T3	
**Total bilirubin**	Range	4~	9~	11~44	
	Median	7	9	13	
	Cases/controls	169/165	156/147	184/197	
	Age adjusted model	1.00 (Ref)	1.03 (0.75–1.42)	0.91 (0.67–1.24)	0.46
	Model 1	1.00 (Ref)	1.09 (0.78–1.54)	0.88 (0.63–1.24)	0.34
	Model 2	1.00 (Ref)	1.02 (0.68–1.54)	0.94 (0.62–1.43)	0.74
**Direct bilirubin**	Range	1~	2~	3~8	
	Median	1	2	3	
	Cases/controls	177/209	214/180	118/120	
	Age adjusted model	1.00 (Ref)	1.46 (1.09–1.97)	1.22 (0.85–1.74)	0.16
	Model 1	1.00 (Ref)	1.41 (1.03–1.93)	1.27 (0.87–1.86)	0.14
	Model 2	1.00 (Ref)	1.68 (1.14–2.47)	1.63 (1.03–2.58)	0.02
**Indirect bilirubin**	Range	3~	8~	9~36	
	Median	6	8	11	
	Cases/controls	237/234	88/79	184/196	
	Age adjusted model	1.00 (Ref)	1.10 (0.77–1.58)	0.93 (0.70–1.23)	0.58
	Model 1	1.00 (Ref)	1.15 (0.78–1.70)	0.91 (0.67–1.24)	0.52
	Model 2	1.00 (Ref)	1.09 (0.68–1.72)	0.97 (0.67–1.40)	0.86

Model 1: adjusted for age, BMI, education level, smoking status, drinking status, physical activity; Model 2: additionally adjusted for alkaline phosphatase, alanine aminotransferase, aspartate aminotransferase, triglyceride, high density lipoprotein, history of hypertension and fasting status.

**Table 4:** Associations of serum bilirubin levels and risk of type 2 diabetes in the cross-sectional analysis of the Dongfeng-Tongji Cohort (n = 15,575).

		Quartiles of serum bilirubin (μmol/L)				*P* for trend
				Q1	Q2		Q3	Q4
**Total bilirubin**	Range	2.8~	10.2~	13.2~	16.9~34.2	
	Median	8.5	11.6	14.8	19.2	
	Cases/n	673/3805	616/3919	602/3917	641/3934	
	Age-, sex- adjusted	1.00 (Ref)	0.84 (0.74–0.95)	0.79 (0.70–0.9)	0.84 (0.74–0.95)	0.01
	Model 1	1.00 (Ref)	0.85 (0.75–0.97)	0.80 (0.70–0.9)	0.84 (0.74–0.95)	0.01
	Model 2	1.00 (Ref)	0.87 (0.76–0.98)	0.83 (0.73–0.94)	0.89 (0.78–1.01)	0.08
	Model 3	1.00 (Ref)	0.90 (0.79–1.02)	0.86 (0.75–0.98)	0.92 (0.80–1.04)	0.19
**Direct bilirubin**	Range	0.8~	3.0~	3.7~	4.6~19.4	
	Median	2.6	3.3	4.1	5.3	
	Cases/n	593/3571	634/3901	667/4097	638/4006	
	Age-, sex- adjusted	1.00 (Ref)	0.94 (0.83–1.06)	0.91 (0.81–1.03)	0.86 (0.75–0.97)	0.02
	Model 1	1.00 (Ref)	0.92 (0.81–1.05)	0.91 (0.80–1.03)	0.83 (0.73–0.95)	0.01
	Model 2	1.00 (Ref)	0.94 (0.82–1.07)	0.94 (0.83–1.08)	0.89 (0.77–1.02)	0.11
	Model 3	1.00 (Ref)	0.99 (0.86–1.13)	1.01 (0.89–1.16)	0.95 (0.83–1.09)	0.51
**Indirect bilirubin**	Range	1.1~	7.1~	9.5~	12.3~27.7	
	Median	5.7	8.1	10.7	14.3	
	Cases/n	678/3874	619/3846	586/3881	649/3974	
	Age-, sex- adjusted	1.00 (Ref)	0.88 (0.78–0.99)	0.79 (0.70–0.90)	0.87 (0.77–0.98)	0.01
	Model 1	1.00 (Ref)	0.88 (0.78–1.00)	0.82 (0.72–0.93)	0.86 (0.76–0.97)	0.02
	Model 2	1.00 (Ref)	0.90 (0.79–1.02)	0.86 (0.75–0.97)	0.90 (0.79–1.02)	0.11
	Model 3	1.00 (Ref)	0.93 (0.81–1.05)	0.88 (0.77–1.00)	0.93 (0.81–1.06)	0.23

Model 1: adjusted for age, sex, body mass index, waist circumference, education level, smoking status, drinking status, physical activity, family history of diabetes, and history of hypertension; Model 2: additionally adjusted for alkaline phosphatase, alanine aminotransferase, and aspartate aminotransferase; Model 3: additionally adjusted for triglyceride and high density lipoprotein.
