# Supplementary material for: Serum bilirubin levels and risk of type 2 diabetes: results from two independent cohorts in middle-aged and elderly Chinese
Source: Sci Rep. 2017 Feb 6;7:41338. doi: 10.1038/srep41338 (PMC5292699; doi:10.1038/srep41338)

**Serum bilirubin levels and risk of type 2 diabetes:** **results from two independent cohorts in middle-aged and elderly Chinese**

Jing Wang^1,2^, Yaru Li^1^, Xu Han^1^, Hua Hu^1^, Fei Wang^1^, Xiulou Li^2^, Kun Yang^2^, Jing Yuan^1^, Ping Yao^1^, Xiaoping Miao^3^, Sheng Wei^3^, Youjie Wang^1^, Weihong Cheng^1^, Yuan Liang^1^, Xiaomin Zhang^1^, Huan Guo^1^, Handong Yang^2^, Jianmin Yuan^4^, Woon-Puay Koh^5^, Frank B. Hu^6^, Tangchun Wu^1^, An Pan^3*^, Meian He^1*^

^1^Department of Occupational and Environmental Health and State Key Laboratory of Environmental Health for Incubating, School of Public Health, Tongji Medical College, Huazhong University of Science and Technology, Wuhan, China

^2^Dongfeng Central Hospital, Dongfeng Motor Corporation and Hubei University of Medicine, Shiyan, Hubei, China

^3^Department of Epidemiology and Biostatistics, School of Public Health, Tongji Medical College, Huazhong University of Science and Technology, Wuhan, China

^4^Division of Cancer Control and Population Sciences, University of Pittsburgh Cancer Institute; and Department of Epidemiology, University of Pittsburgh Graduate School of Public Health, Pittsburgh, PA

^5^Duke-NUS Medical School, Singapore

^6^Department of Nutrition and Department of Epidemiology, Harvard T.H. Chan School of Public Health, Boston, MA, USA

*** Correspondence:** Meian He or An Pan

**Meian He**: Department of Occupational and Environmental Health and State Key Laboratory of Environmental Health for Incubatiing, School of Public Health, Tongji Medical College, Huazhong University of Science and Technology,13 Hangkong Rd, Wuhan 430030, Hubei, China. Tel: 86-27-83657914; Fax: 86-27-83692560; E-mail: hemeian@hotmail.com

**An Pan:** Department of Epidemiology and Biostatistics, School of Public Health, Tongji Medical College, Huazhong University of Science and Technology, 13 Hangkong Rd, Wuhan 430030, Hubei, China. Tel: 86-27-83627309; Fax: 86-27-83627309;

E-mail: [panan@hust.edu.cn](mailto:panan@hust.edu.cn)

**SUPPLEMENTARY INFORMATION**

1. **Supplementary tables**
2. **Supplementary figure**

**1. Supplementary Tables**

Supplementary Table S1. Associations of serum bilirubin levels and risk of type 2 diabetes incidence among the participants without impaired fasting glucose at baseline (n= 6105)

Supplementary Table S2. Associations of serum bilirubin levels and risk of type 2 diabetes incidence stratified by smoking status in SCHS

Supplementary Table S3. Associations of serum bilirubin levels with risk of type 2 diabetes in cross-sectional analysis among different populations (diabetes with different duration)

Supplementary Table S4. Baseline characteristics of the subjects in the Singapore Chinese Health Study

**2. Supplementary Figure**

Supplementary Figure S1. Stratified analyses of the association between serum bilirubin concentrations and type 2 diabetes risk

**Supplementary Table S1. Associations of serum bilirubin levels and risk of type 2 diabetes incidence** **among the participants without impaired fasting glucose at baseline (n= 6105)**

|  |  | **Quartiles of serum bilirubin (μmol/L)** | | | | ***P* for trend** |
| --- | --- | --- | --- | --- | --- | --- |
|  |  | **Q1** | **Q2** | **Q3** | **Q4** |  |
| **Total bilirubin** | Range | 2.8~ | 10.2~ | 13.1~ | 16.6~34.1 |  |
|  | Median | 8.5 | 11.6 | 14.7 | 19.1 |  |
|  | Cases/Person-years | 53/6955.11 | 37/7156.06 | 53/7056.51 | 43/7100.37 |  |
|  | Age-, sex- adjusted | 1.00 (Ref) | 0.72 (0.47-1.10) | 0.99 (0.67-1.45) | 0.80 (0.53-1.21) | 0.53 |
|  | Model 1 | 1.00 (Ref) | 0.70 (0.46-1.07) | 1.01 (0.69-1.50) | 0.81 (0.54-1.24) | 0.64 |
|  | Model 2 | 1.00 (Ref) | 0.68 (0.44-1.05) | 1.05 (0.71-1.55) | 0.75 (0.49-1.15) | 0.45 |
| **Direct bilirubin** | Range | 0.8~ | 3.0~ | 3.7~ | 4.5~17.6 |  |
|  | Median | 2.6 | 3.3 | 4.1 | 5.2 |  |
|  | Cases/Person-years | 45/6891.98 | 52/7233.07 | 38/6637.59 | 51/7505.42 |  |
|  | Age-, sex- adjusted | 1.00 (Ref) | 1.18 (0.79-1.76) | 0.95 (0.61-1.46) | 1.03 (0.68-1.57) | 0.86 |
|  | Model 1 | 1.00 (Ref) | 1.20 (0.80-1.81) | 1.01 (0.65-1.56) | 1.12 (0.73-1.71) | 0.81 |
|  | Model 2 | 1.00 (Ref) | 1.18 (0.78-1.78) | 1.02 (0.65-1.59) | 1.09 (0.70-1.67) | 0.90 |
| **Indirect bilirubin** | Range | 1.1~ | 7.0~ | 9.4~ | 12.2~27.5 |  |
|  | Median | 5.6 | 8 | 10.5 | 14.1 |  |
|  | **Cases/Person-years** | 49/6777.49 | 42/7150.83 | 48/7153.45 | 47/7186.29 |  |
|  | Age-, sex- adjusted | 1.00 (Ref) | 0.83 (0.55-1.25) | 0.96 (0.65-1.44) | 0.89 (0.60-1.34) | 0.77 |
|  | Model 1 | 1.00 (Ref) | 0.82 (0.54-1.25) | 1.03 (0.69-1.54) | 0.90 (0.60-1.37) | 0.87 |
|  | Model 2 | 1.00 (Ref) | 0.82 (0.53-1.26) | 1.08 (0.72-1.63) | 0.85 (0.56-1.29) | 0.68 |

Model 1: adjusted for age, sex, BMI, waist circumference, education level, smoking status, drinking status, physical activity, and family history of diabetes;

Model 2: additionally adjusted for serum levels of alkaline phosphatase, alanine aminotransferase, and aspartate aminotransferase, triglyceride, high density lipoprotein and history of hypertension.

**Supplementary Table S2. Associations of serum bilirubin levels and risk of type 2 diabetes incidence stratified by smoking status in SCHS**

| **Bilirubin** | **Smoking status** | | ***P* _interaction_** |
| --- | --- | --- | --- |
|  | **Never** | **Ever** |  |
| Total bilirubin | 1.01 (0.95-1.08) | 0.94 (0.81-1.09) | 0.29 |
| Direct bilirubin | 1.25 (0.98-1.60) | 0.75 (0.45-1.27) | 0.11 |
| Indirect bilirubin | 0.99 (0.92-1.07) | 0.94 (0.80-1.12) | 0.46 |

Assigned the median value to each tertile, and treated it as continuous variable in the model. Adjusted for age, BMI, education level, drinking status, physical activity, alkaline phosphatase, alanine aminotransferase, aspartate aminotransferase, history of hypertension, triglyceride, high density lipoprotein, and fasting.

**Supplementary Table S**3. Associations of serum bilirubin levels with risk of type 2 diabetes in cross-sectional analysis among different populations (diabetes with different duration)

| **Bilirubin types** | | **Odds ratio (95% confidence interval) per SD** | ***P*** |
| --- | --- | --- | --- |
| **Diabetics with duration less and equal than 1 year vs. normal glucose individuals** | | | |
| Total bilirubin | 1.08 (1.01-1.16) | | 0.03 |
| Direct bilirubin | 1.10 (1.02-1.18) | | 0.01 |
| Indirect bilirubin | 1.10 (1.01-1.54) | | 0.04 |
| **Diabetics with duration more than 1 years vs. normal glucose individuals .** | | | |
| Total bilirubin | 0.93 (0.87-0.99) | | 0.03 |
| Direct bilirubin | 0.97 (0.90-1.03) | | 0.29 |
| Indirect bilirubin | 0.93 (0.87-0.99) | | 0.03 |

All models adjusted for age, sex, BMI, waist circumference, education level, smoking status, drinking status, physical activity, family history of diabetes, history of hypertension, serum levels of alkaline phosphatase, alanine aminotransferase, aspartate aminotransferase; triglyceride and high density lipoprotein.

**Supplementary Table S4. Baseline characteristics of the subjects in the Singapore Chinese Health Study**

| **Variable** | **Overall** | **Non-diabetic** | **Diabetic** | ***P*** |
| --- | --- | --- | --- | --- |
|  | **(n = 1018)** | **(n = 509)** | **(n = 509)** |  |
| Total bilirubin (μmol/L) | 9.0 (8.0, 12.0) | 9.0 (8.0, 12.0) | 9.0 (8.0, 12.0) | 0.75 |
| Direct bilirubin (μmol/L ) | 2.0 (1.0, 2.0) | 2.0 (1.0, 2.0) | 2.0 (1.0, 2.0) | 0.25 |
| Indirect bilirubin (μmol/L) | 8.0 (6.0, 10.0) | 8.0 (6.0, 10.0) | 8.0 (6.0, 10.0) | 0.57 |
| Age, years | 59.7 (6.2) | 59.7 (6.3) | 59.6 (6.2) | 0.77 |
| Sex, men（%） | 41.7 | 41.7 | 41.7 | 1.00 |
| High school or above, (%) | 38.9 | 40.3 | 37.5 | 0.37 |
| Smoker, (%) | 27.2 | 25.9 | 28.5 | 0.36 |
| Drinker, (%) | 13 | 13.4 | 12.6 | 0.71 |
| Physical activity, (%) | 20 | 19.8 | 20.2 | 0.88 |
| HbA1c | 6.2 (1.2) | 5.5 (0.3) | 6.8 (1.4) | < 0.001 |
| ALP (U/L) | 81.0 (67.0, 96.0) | 77.0 (67.0, 90.0) | 84.0 (67.0, 101.0) | < 0.001 |
| ALT (U/L) | 23.0 (17.0, 32.0) | 20.0 (15.0, 27.0) | 27.0 (20.0, 37.0) | < 0.001 |
| AST (U/L) | 25.0 (21.0, 30.0) | 24.0 (21.0, 29.0) | 26.0 (21.0, 32.0) | < 0.001 |
| Total cholesterol (mmol/L) | 5.3 (0.9) | 5.2 (0.9) | 5.3 (0.9) | 0.14 |
| Triglyceride (mmol /L) | 1.8 (1.2, 2.7) | 1.6 (1.1, 2.2) | 2.2 (1.5, 3.0) | < 0.001 |
| High density lipoprotein (mmol/L) | 1.2 (0.3) | 1.2 (0.3) | 1.1 (0.2) | < 0.001 |
| Low density lipoprotein (mmol/L) | 3.2 (0.8) | 3.2 (0.8) | 3.2 (0.9) | 0.86 |
| Overweight/obesity, (%) | 44.9 | 32.8 | 57.0 | < 0.001 |
| Hypertension, (%) | 36.1 | 25.3 | 46.8 | < 0.001 |

Data are means (SD), percentage (%), or median (interquartile range); **P*-value was calculated after adjustment for age, sex except for itself for comparison incident diabetes with non-diabetes group.

ALP, alkaline phosphatase; ALT, alanine aminotransferase; AST, aspartate aminotransferase.

**Figure legend**

**Supplementary Figure S1. Stratified analyses of the association between serum bilirubin concentrations and type 2 diabetes risk**

Adjusted for age, sex, BMI, education level, smoking status, drinking status, physical activity, family history of diabetes, history of hypertension, liver function (alkaline phosphatase, alanine aminotransferase and aspartate aminotransferase), triglyceride, high density lipoprotein, except for the stratifying factor in the corresponding analysis.


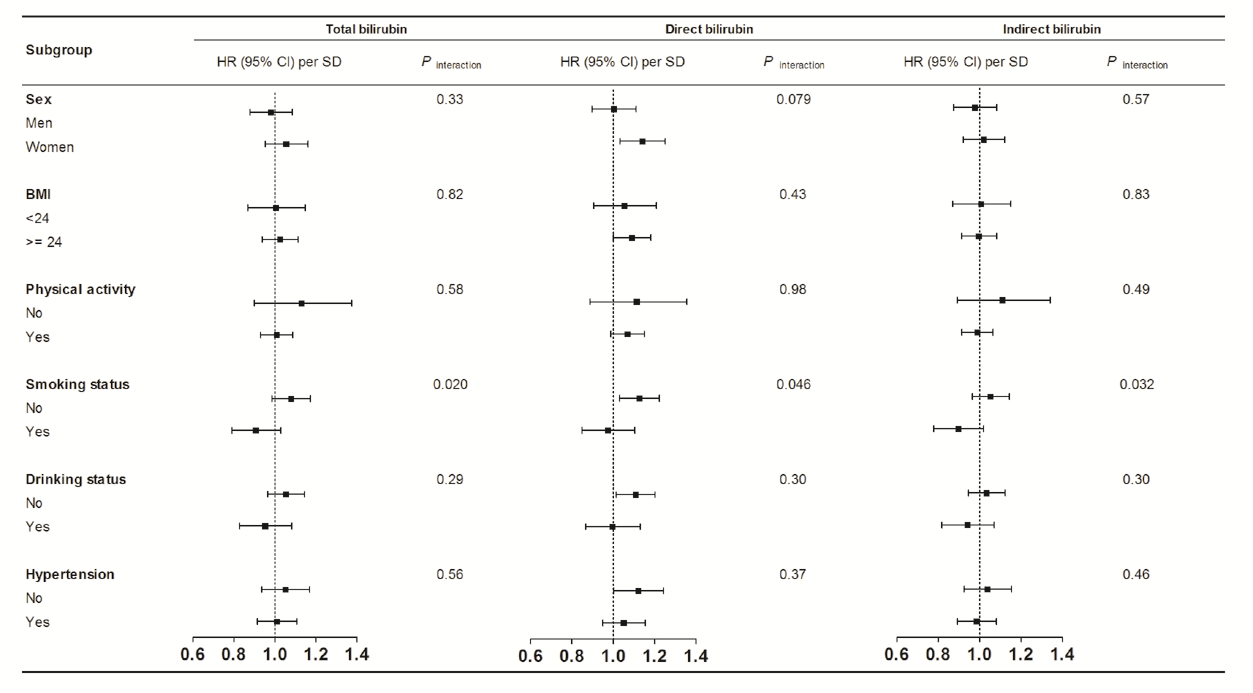

Supplement: Supplementary Tables and Figure [file srep41338-s1.doc]
